# Prognostic Implications of Vancomycin Therapeutic Drug Monitoring for Critically Ill Stroke Patients: Evidence From a Subtype‐Oriented Analysis

**DOI:** 10.1002/cns.70799

**Published:** 2026-02-19

**Authors:** Hongbo San, Jing Feng, Hongyu Zhang, Mengsi Zhan, Jiahao Yao, Jianheng Gu, Lang Li, Yihan Hu, Chenxing Liu, Haonan Chen, Jianing Jiang, Shuang Liu, Xuefeng Wang

**Affiliations:** ^1^ Department of Neurosurgery The Fourth Affiliated Hospital of Harbin Medical University Harbin China; ^2^ Plastic Surgery Hospital Chinese Academy of Medical Sciences and Peking Union Medical College Beijing China; ^3^ School of Basic Medicine Harbin Medical University Harbin China

**Keywords:** MIMIC‐IV, mortality, stroke, therapeutic drug monitoring, vancomycin

## Abstract

**Background:**

Post‐stroke infections, particularly those caused by Gram‐positive pathogens, are frequent in neurocritical care and worsen outcomes. Vancomycin is widely used against severe Gram‐positive infections, and therapeutic drug monitoring (TDM) is recommended to optimize efficacy and minimize toxicity. However, its prognostic value in critically ill stroke patients remains unclear.

**Methods:**

Clinical data were obtained from the MIMIC‐IV (v3.1) database. Adult patients with ischemic or hemorrhagic stroke receiving intravenous vancomycin were included. Patients were stratified by stroke subtype and TDM status. Propensity score matching (PSM) was performed to balance baseline characteristics. The primary outcome was 28‐day mortality; secondary outcomes included ICU, hospital, 60‐, 90‐, and 365‐day mortality.

**Results:**

A total of 1266 patients were analyzed (543 hemorrhagic, 723 ischemic). In hemorrhagic stroke, 28‐day mortality was lower in the TDM group (24.6% vs. 48.2%, *p* < 0.001), and the association persisted after PSM (23.3% vs. 40.8%, *p* = 0.007). Multivariable Cox analysis confirmed TDM as an independent predictor of reduced 28‐day mortality (adjusted HR = 0.29, 95% CI 0.15–0.56, *p* < 0.001). No significant association was observed in ischemic stroke.

**Conclusions:**

Vancomycin TDM was independently linked to improved survival in hemorrhagic but not ischemic stroke, supporting individualized antimicrobial optimization in neurocritical care.

## Graphical abstract

Using the MIMIC‐IV critical care database, vancomycin therapeutic drug monitoring was independently associated with improved short‐ and long‐term survival in patients with hemorrhagic stroke, but not ischemic stroke. These findings highlight the value of individualized antimicrobial optimization in neurocritical care.

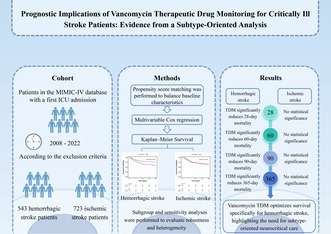

**Abbreviations**
APS III: Acute Physiology Score III
ARC: Augmented Renal Clearance
ASDs: absolute standardized differences
AUC/MIC: Area Under the Concentration–Time Curve to Minimum Inhibitory Concentration ratio
BIDMC: Beth Israel Deaconess Medical Center
BUN: blood urea nitrogen
CCI: Charlson Comorbidity Index
CI/CIs: Confidence Interva(s)
CITI: Collaborative Institutional Training Initiative
CKD‐EPI: Chronic Kidney Disease Epidemiology Collaboration
CNS: central nervous system
DALYs: Disability‐Adjusted Life Years
ERC: Enhanced Renal Clearance
GBD: Global Burden of Disease
GFR: Glomerular Filtration Rate
HR/HRs: Hazard Ratio(s)
HS: hemorrhagic stroke
ICD‐9/10: International Classification of Diseases, Ninth and Tenth Revisions
ICU: intensive care unit
IQR/IQRs: Interquartile Range(s)
IS: ischemic stroke
MIMIC‐IV: Medical Information Mart for Intensive Care IV
MIT: Massachusetts Institute of Technology
OASIS: Oxford Acute Severity of Illness Score
*P*
: Probability value
PSM: Propensity Score Matching
RRT: renal replacement therapy
SAP: Stroke‐Associated Pneumonia
SAPS II: Simplified Acute Physiology Score II
SDI: Sociodemographic Index
SIDS: Stroke‐Induced Immunosuppression
SOFA: Sequential Organ Failure Assessment
SQL: Structured Query Language
TDM: therapeutic drug monitoring

## 1 Introduction

The Global Burden of Disease (GBD) 2021 report identifies stroke as the world's third most common cause of death and the fourth major contributor to disability‐adjusted life years (DALYs) [1]. Countries with lower sociodemographic index (SDI) values—mainly low‐ and middle‐income regions—bear a disproportionately high share of the global stroke burden [1]. As most neurological diseases increase in incidence with advancing age, the ongoing global population aging—together with overall population growth—is expected to further exacerbate the burden of these disorders [2, 3, 4]. Ischemic stroke (IS), the predominant subtype of cerebrovascular events, occurs when cerebral blood vessels become transiently or permanently obstructed [5, 6, 7]. In contrast, hemorrhagic strokes (HS)—accounting for approximately 10% to 40% of all cases depending on regional variations—arise from the rupture of intracranial arteries [8, 9].

Despite substantial progress in the management of acute stroke, infectious complications remain a major clinical challenge, with approximately one‐third of patients experiencing pulmonary infections during their clinical course [10, 11, 12]. Post‐stroke infections usually result from a multifactorial interplay involving neurological injury, stroke‐induced immunosuppression, and impairment of host defense mechanisms [13]. Increasing evidence indicates that post‐stroke infections, particularly stroke‐associated pneumonia (SAP), are closely associated with poor neurological recovery, increased mortality, and unfavorable long‐term outcomes [12, 14, 15, 16]. Due to stroke‐related respiratory dysfunction, about 6% of IS and nearly 30% of HS patients require endotracheal intubation [17]. Although mechanical ventilation is essential for maintaining adequate respiration, it also increases the risk of secondary pulmonary infections. Evidence suggests that respiratory specimens from over half of ventilator‐associated pneumonia cases yield Gram‐positive cocci [18]. Likewise, Gram‐positive organisms are frequently implicated in post‐stroke pneumonia, and the proportion of methicillin‐resistant *Staphylococcus aureus* (MRSA) among these pathogens has shown a steady rise in recent years [19, 20]. Previous reports have documented that MRSA bacteremia carries a 30‐day mortality rate of up to 30%, highlighting its severe clinical consequences [21, 22]. Therefore, early identification of high‐risk patients and optimized antimicrobial management are essential to improve clinical outcomes in this vulnerable population.

Vancomycin, a glycopeptide antibiotic with potent activity against Gram‐positive pathogens such as MRSA, continues to serve as a key treatment for patients with severe infections in critical care settings [23]. Current guidelines recommend vancomycin therapeutic drug monitoring (TDM) to achieve individualized dosing and maintain an optimal AUC/MIC ratio of 400–600 mg·h/L, ensuring efficacy while minimizing nephrotoxicity risk [24]. Previous studies have demonstrated that TDM significantly improves target attainment and reduces nephrotoxicity in general ICU and sepsis population [25, 26]. Moreover, several investigations have suggested that TDM implementation may significantly reduce mortality in critically ill patients [27, 28]. However, evidence regarding the role of TDM in critically ill stroke populations remains extremely limited. Stroke‐related pathophysiological alterations—such as fluctuating renal function and systemic inflammatory responses—may substantially influence the pharmacokinetic profile of vancomycin [29]. It is still unclear whether applying vancomycin TDM improves survival outcomes among critically ill patients suffering from HS or IS, or if its clinical impact varies between these two stroke types [27, 30, 31]. Accordingly, we utilized data from the large MIMIC‐IV critical care database to investigate the association of vancomycin TDM with mortality among critically ill individuals suffering from HS or IS. We hypothesized that TDM‐guided vancomycin dosing would be associated with reduced mortality, with potentially different effects between the two stroke subtypes. If confirmed, these findings would provide evidence supporting the clinical value of vancomycin TDM, thereby promoting optimized and individualized antimicrobial management and improved outcomes in critically ill stroke patients.

## 2 Methods

### 2.1 Data Source

Data for this study were drawn from MIMIC‐IV (v3.1), a public critical‐care database curated by the Laboratory for Computational Physiology at the Massachusetts Institute of Technology (MIT) [32]. MIMIC‐IV includes comprehensive, de‐identified data on patients from Beth Israel Deaconess Medical Center (BIDMC) who were admitted to its intensive care units (ICUs) between 2008 and 2022. The database has been authorized for use by the Institutional Review Boards (IRBs) of MIT and BIDMC, and because all data are anonymized, informed consent was not required. The investigator (Hongbo San; CITI Certificate ID 67554259) completed human‐subjects training and was granted access. This analysis follows the principles of the Declaration of Helsinki and qualifies for IRB exemption as it relies solely on publicly available, de‐identified data.

### 2.2 Study Population

**FIGURE 1:**
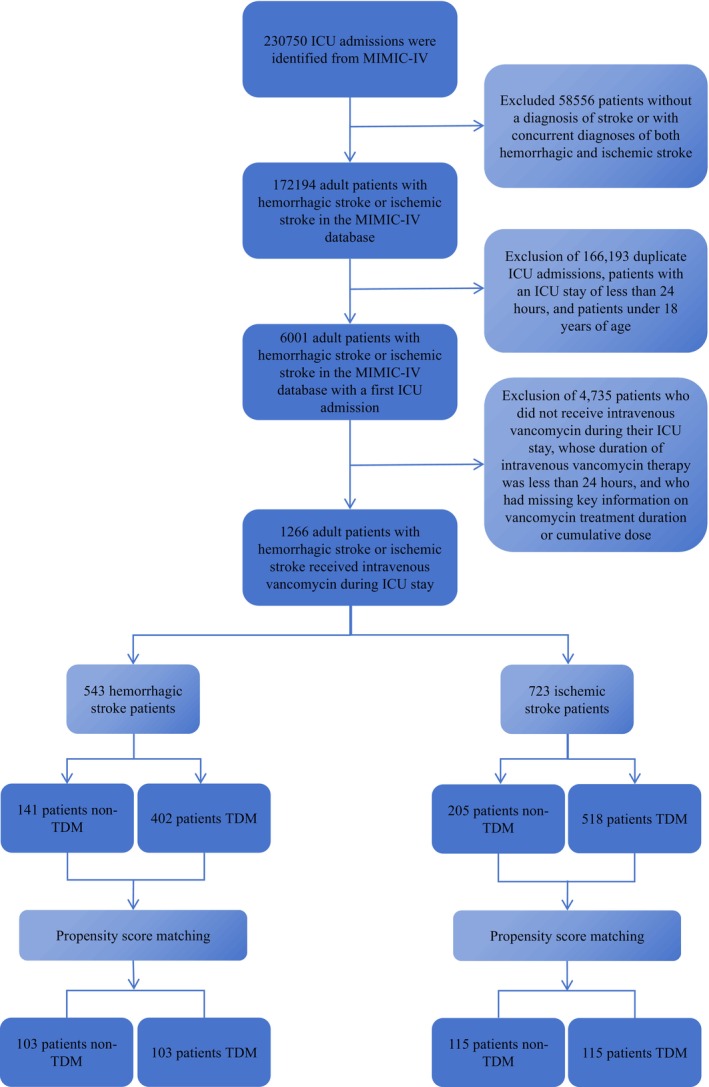
Flowchart of patient selection from the MIMIC‐IV database.

Patient selection was determined using the International Classification of Diseases, Ninth and Tenth Revisions (ICD‐9/10), to identify individuals diagnosed with IS or HS who received intravenous vancomycin therapy during or around their ICU stay. The exclusion criteria were as follows: (1) absence of an ICD‐9/10 diagnosis consistent with IS or HS; (2) concurrent diagnoses of both IS and HS; (3) multiple ICU admissions, retaining only data from the initial stay; (4) ICU length of stay shorter than 24 h; (5) age under 18 years at the time of first ICU admission; (6) lack of intravenous vancomycin administration during the ICU stay; (7) total duration of intravenous vancomycin therapy shorter than 24 h; and (8) missing data on vancomycin treatment duration or cumulative dose. Based on whether TDM for vancomycin was performed—defined as at least one plasma concentration measurement (trough, peak, or random)—the study cohort was stratified into non‐TDM (control) and TDM (experimental) groups (Figure 1).

### 2.3 Data Collection

Using Structured Query Language (SQL) queries, clinical data were extracted from the MIMIC‐IV database. The analysis incorporated multiple variables, including demographic data, physiological indicators, laboratory indicators, comorbid conditions, therapeutic procedures, and disease severity indices, as well as information on vancomycin administration and TDM. Using the CKD‐EPI formula, the estimated glomerular filtration rate (eGFR) was estimated [33]. Detailed variable definitions are provided in Table S1.

### 2.4 Clinical Outcomes

28‐day mortality was defined as the primary outcome, while secondary endpoints comprised ICU and hospital, and overall mortality at 60‐, 90‐, and 365‐day follow‐ups.

### 2.5 Statistical Analysis

Variables exceeding 20% missingness were removed to reduce potential bias, whereas those with less than 20% missingness were imputed using a random forest algorithm (trained on other non‐missing variables) implemented in the “mice” package of R software to ensure data completeness (Table S2) [34, 35]. Continuous variables were expressed as medians with interquartile ranges (IQRs), and categorical variables were presented as counts and percentages. To compare groups, Kruskal–Wallis and Wilcoxon rank‐sum tests were applied to continuous variables, whereas chi‐square and Fisher's exact tests were used for categorical variables.

Participants were classified into two cohorts according to stroke subtype: HS and IS. Within each cohort, patients were further categorized into TDM and non‐TDM groups according to whether vancomycin serum concentrations were measured during ICU stay. Propensity score matching (PSM) was performed within each cohort to minimize potential selection bias between the TDM and non‐TDM groups. A 1:1 nearest‐neighbor PSM was performed with a caliper of 0.1 using the MatchIt package in R to ensure comparability of baseline characteristics.

The association between TDM and mortality was analyzed through Cox proportional hazards regression, with results presented as hazard ratios (HRs) and corresponding 95% confidence intervals (CIs). To account for differences in treatment intensity, vancomycin medication time and accumulated dose were included in the fully adjusted Cox model (Model 3). Kaplan–Meier survival curves were plotted to illustrate survival differences, which were compared using the log‐rank test.

Subgroup analyses were conducted within each cohort based on demographic and clinical characteristics, including age, sex, race, renal function, sepsis, mechanical ventilation, and disease severity scores, including the Oxford Acute Severity of Illness Score (OASIS), Acute Physiology Score III (APS III), and Simplified Acute Physiology Score II (SAPS II).

To rigorously address potential biases—particularly immortal time bias and surveillance bias—and to verify the robustness of our primary findings, we conducted five pre‐specified sensitivity analyses. For each sensitivity scenario, we repeated the propensity score matching procedure and performed Cox proportional hazards regression analyses on both the pre‐match and post‐match cohorts. The models were defined as follows: Model 1 excluded patients with a survival time of less than 48 h to minimize immortal time bias introduced by early deaths. Model 2 excluded patients whose intravenous vancomycin treatment duration was less than 48 h to ensure all subjects had a sufficient window for potential monitoring. Model 3 excluded patients with positive microbiological cultures for MRSA to minimize confounding due to pathogen‐specific treatment responses. Model 4 further adjusted for the overall intensity of care by including the total laboratory test count and blood culture frequency during the ICU stay. Finally, Model 5 applied a stricter definition of TDM, classifying patients into the TDM group only if they had at least two measured vancomycin serum concentrations, thereby reducing potential misclassification from random single measurements.

All statistical tests were two‐tailed, and a *p*‐value < 0.05 was considered statistically significant. All analyses were performed using R software (version 4.2.2).

## 3 Results

### 3.1 Baseline Characteristics of Stroke Patients

Before matching, baseline characteristics differed significantly between patients with and without vancomycin TDM in both stroke subtypes. In the HS cohort, patients undergoing TDM exhibited higher body weight, lower serum calcium levels, and a higher prevalence of hypertension, renal disease, sepsis, and MRSA infection. They also required mechanical ventilation and renal replacement therapy (RRT) more frequently, utilized vasopressors less often, and had significantly higher vancomycin accumulated dose and longer medication time. In the IS cohort, patients receiving TDM were younger, predominantly male, and had higher body weight, heart rate, and blood urea nitrogen (BUN) levels. Clinical comparisons revealed a higher prevalence of coagulopathy, sepsis, and septic shock, but a lower prevalence of heart failure and coronary artery disease in the TDM group. Additionally, the TDM group presented with higher Sequential Organ Failure Assessment (SOFA), OASIS, and APS III scores, lower Charlson Comorbidity Index (CCI) scores, and a higher requirement for vasopressors, mechanical ventilation, and RRT. Similarly, vancomycin accumulated dose and medication time were significantly higher in the TDM group (Table S3).

After performing separate 1:1 PSM within each cohort, 103 matched pairs were obtained for the HS group and 115 matched pairs for the IS group. The matching process effectively balanced the majority of baseline characteristics, achieving absolute standardized differences (ASDs) below 0.1 across demographics, comorbidities, laboratory indices, and severity scores (all *p* > 0.05) (Table 1; Figures S1 and S2). This indicates that potential selection bias was substantially minimized for these covariates. However, vancomycin medication duration and accumulated dose remained imbalanced (ASD > 0.1), with the TDM group exhibiting significantly higher values despite matching. To rigorously control for this residual imbalance and the potential confounding effect of treatment intensity, both vancomycin medication duration and accumulated dose were explicitly incorporated as covariates in the fully adjusted multivariable Cox regression model (Model 3).

**TABLE 1:** Baseline characteristics and demographics of hemorrhagic and ischemic stroke patients after propensity score matching.

Characteristic	Hemorrhagic stroke (*n* = 206)			Ischemic stroke patient (*n* = 230)		
	Non‐TDM group (*n* = 103^a^)	TDM group (*n* = 103^a^)	*p* ^b^	Non‐TDM group (*n* = 115)	TDM group (*n* = 115)	*p* ^b^
Age	63 (54, 80)	64 (55, 76)	0.918	68 (55, 80)	69 (58, 79)	0.682
Gender (*n*, %)			0.674			0.51
Male	55 (53.4)	58 (56.3)		56 (48.7)	61 (53.0)	
Female	48 (46.6)	45 (43.7)		59 (51.3)	54 (47.0)	
Race (*n*, %)			0.486			0.894
White	53 (51.5)	48 (46.6)		65 (56.5)	66 (57.4)	
Other	50 (48.5)	55 (53.4)		50 (43.5)	49 (42.6)	
*Vital signs*						
Weight (Kg)	76 (64, 87)	74 (61, 88)	0.56	78 (69, 87)	74 (64, 94)	0.792
Heart rate (bpm)	83 (71, 93)	82 (74, 96)	0.479	85 (71, 94)	80 (72, 94)	0.455
Respiratory rate(/min)	19.0 (16.0, 21.0)	19.0 (16.0, 23.0)	0.514	20.0 (17.0, 24.0)	20.0 (17.0, 23.0)	0.721
MAP (mmHg)	83 (73, 94)	82 (70, 94)	0.708	79 (68, 93)	81 (70, 94)	0.275
Temperature (°C)	37.06 (36.61, 37.61)	37.06 (36.61, 37.44)	0.629	37.00 (36.67, 37.33)	36.89 (36.72, 37.28)	0.573
SO_2_ (%)	88.00 (85.00, 88.00)	88.00 (85.00, 88.00)	0.96	85.00 (85.00, 88.00)	85.00 (85.00, 88.00)	0.941
*Laboratory tests*						
Hematocrit (%)	36 (32, 39)	35 (31, 39)	0.429	33 (28, 39)	33 (29, 38)	0.759
Hemoglobin (g/dL)	11.80 (10.50, 13.30)	11.60 (10.20, 12.90)	0.428	10.90 (9.00, 12.70)	11.10 (8.90, 12.60)	0.792
WBC (k/uL)	11.4 (9.2, 14.3)	12.2 (9.7, 15.5)	0.37	12 (9, 16)	12 (8, 15)	0.431
Platelets (K/uL)	200 (155, 273)	218 (167, 267)	0.584	198 (143, 268)	192 (123, 268)	0.459
Glu (mg/dL)	149 (115, 189)	141 (122, 180)	0.761	136 (111, 184)	136 (110, 187)	0.95
HCO3 (mEg/L)	23.0 (21.0, 25.0)	23.0 (21.0, 25.0)	0.89	22.0 (20.0, 24.0)	23.0 (20.0, 25.0)	0.694
Ca (mg/dL)	8.60 (8.10, 9.10)	8.60 (8.20, 9.10)	0.891	8.40 (7.90, 8.70)	8.50 (7.90, 8.90)	0.495
K (mEg/L)	3.90 (3.60, 4.30)	3.90 (3.60, 4.40)	0.991	4.00 (3.70, 4.50)	4.20 (3.80, 4.50)	0.31
BUN (mg/dL)	16 (11, 26)	17 (11, 24)	0.545	20 (13, 28)	21 (14, 33)	0.472
eGFR (mL/min/1.73m^2^)	79 (55, 101)	83 (57, 101)	0.664	69 (46, 92)	75 (49, 90)	0.934
*Comorbidity diseases (n, %)*						
Heart failure (*n*, %)			0.638			0.886
No	92 (89.3)	94 (91.3)		80 (69.6)	79 (68.7)	
Yes	11 (10.7)	9 (8.7)		35 (30.4)	36 (31.3)	
Coronary artery disease (*n*, %)			0.367			0.654
No	90 (87.4)	94 (91.3)		86 (74.8)	83 (72.2)	
Yes	13 (12.6)	9 (8.7)		29 (25.2)	32 (27.8)	
Cardiac arrhythmias (*n*, %)			> 0.999			> 0.999
No	99 (96.1)	98 (95.1)		114 (99.1)	113 (98.3)	
Yes	4 (3.9)	5 (4.9)		1 (0.9)	2 (1.7)	
Diabetes (*n*, %)			0.873			0.788
No	77 (74.8)	76 (73.8)		70 (60.9)	68 (59.1)	
Yes	26 (25.2)	27 (26.2)		45 (39.1)	47 (40.9)	
Hypertension (*n*, %)			0.725			0.868
No	84 (81.6)	82 (79.6)		92 (80.0)	93 (80.9)	
Yes	19 (18.4)	21 (20.4)		23 (20.0)	22 (19.1)	
Renal disease (*n*, %)			0.8			0.516
No	95 (92.2)	94 (91.3)		93 (80.9)	89 (77.4)	
Yes	8 (7.8)	9 (8.7)		22 (19.1)	26 (22.6)	
PVD (*n*, %)			> 0.999			0.27
No	101 (98.1)	101 (98.1)		110 (95.7)	106 (92.2)	
Yes	2 (1.9)	2 (1.9)		5 (4.3)	9 (7.8)	
Coagulopathy (*n*, %)			> 0.999			0.525
No	87 (84.5)	87 (84.5)		101 (87.8)	104 (90.4)	
Yes	16 (15.5)	16 (15.5)		14 (12.2)	11 (9.6)	
SEPSIS (*n*, %)			0.818			0.42
No	10 (9.7)	11 (10.7)		16 (13.9)	12 (10.4)	
Yes	93 (90.3)	92 (89.3)		99 (86.1)	103 (89.6)	
Septic shock (*n*, %)			0.447			0.276
No	93 (90.3)	96 (93.2)		100 (87.0)	94 (81.7)	
Yes	10 (9.7)	7 (6.8)		15 (13.0)	21 (18.3)	
*Therapy (n, %)*						
Vasopressin (*n*, %)			0.603			0.677
No	96 (93.2)	94 (91.3)		101 (87.8)	103 (89.6)	
Yes	7 (6.8)	9 (8.7)		14 (12.2)	12 (10.4)	
Mechanical ventilation (*n*, %)			> 0.999			0.45
No	73 (70.9)	73 (70.9)		88 (76.5)	83 (72.2)	
Yes	30 (29.1)	30 (29.1)		27 (23.5)	32 (27.8)	
RRT (*n*, %)			> 0.999			0.604
No	100 (97.1)	99 (96.1)		108 (93.9)	106 (92.2)	
Yes	3 (2.9)	4 (3.9)		7 (6.1)	9 (7.8)	
*Severity of illness scores*						
SOFA score	3.00 (2.00, 6.00)	4.00 (2.00, 5.00)	0.779	5.0 (3.0, 7.0)	5.0 (3.0, 8.0)	0.562
OASIS	34 (29, 40)	35 (30, 40)	0.384	35 (31, 40)	36 (29, 42)	0.913
APS III	39 (27, 53)	40 (29, 57)	0.524	47 (33, 62)	48 (39, 61)	0.449
SAPS II	36 (27, 46)	37 (30, 44)	0.626	39 (31, 48)	39 (33, 50)	0.562
CCI	6.00 (3.00, 7.00)	5.00 (4.00, 7.00)	0.469	7.00 (4.00, 9.00)	7.00 (5.00, 9.00)	0.54
*Infectious pathogen, n (%)*						
MRSA (*n*, %)			> 0.999			0.518
No	101 (98.1)	102 (99.0)		111 (96.5)	109 (94.8)	
Yes	2 (1.9)	1 (1.0)		4 (3.5)	6 (5.2)	
*Details of first administration of vancomycin in ICU*						
Accumulated dose (g)	2.00 (1.50, 3.00)	2.00 (2.00, 3.00)	0.043	2.00 (1.00, 3.00)	2.00 (1.00, 2.75)	0.899
Medication time (d)	2.17 (1.58, 3.17)	2.29 (1.67, 3.04)	0.807	2.08 (1.54, 2.67)	2.00 (1.50, 2.92)	0.96
*Clinical outcomes, n (%)*						
ICU mortality (*n*, %)			0.021			0.389
No	77 (74.8)	90 (87.4)		92 (80.0)	97 (84.3)	
Yes	26 (25.2)	13 (12.6)		23 (20.0)	18 (15.7)	
Hospital mortality (*n*, %)			0.004			0.337
No	76 (73.8)	92 (89.3)		93 (80.9)	87 (75.7)	
Yes	27 (26.2)	11 (10.7)		22 (19.1)	28 (24.3)	
28‐day mortality (*n*, %)			0.007			0.334
No	61 (59.2)	79 (76.7)		78 (67.8)	71 (61.7)	
Yes	42 (40.8)	24 (23.3)		37 (32.2)	44 (38.3)	
60‐day mortality (*n*, %)			0.009			0.139
No	58 (56.3)	76 (73.8)		74 (64.3)	63 (54.8)	
Yes	45 (43.7)	27 (26.2)		41 (35.7)	52 (45.2)	
90‐day mortality (*n*, %)			0.003			0.184
No	52 (50.5)	73 (70.9)		69 (60.0)	59 (51.3)	
Yes	51 (49.5)	30 (29.1)		46 (40.0)	56 (48.7)	
365‐day mortality (*n*, %)			0.008			0.291
No	43 (41.7)	62 (60.2)		62 (53.9)	54 (47.0)	
Yes	60 (58.3)	41 (39.8)		53 (46.1)	61 (53.0)	

Abbreviations: APS III, Simplified Acute Physiology Score III; BUN, blood urea nitrogen; Ca, Calcium; CCI, Charlson Comorbidity Index; eGFR, estimated glomerular filtration rate; Glu, glutamate; ICU, intensive care unit; MAP, mean arterial pressure; MRSA, Methicillin‐resistant *Staphylococcus aureus*; OASIS, Oxford Acute Severity of Illness Score; PVD, peripheral vascular disease; RRT, renal replacement therapy; SAPS II, Simplified Acute Physiology Score II; SOFA score, Sequential Organ Failure Assessment score; SpO_2_, peripheral oxygen saturation; WBC, white blood cell.

^a^ IQR; *n* (%).

^b^ Wilcoxon rank sum test; Pearson's Chi‐squared test; Fisher's exact test.

### 3.2 Clinical Outcomes

In the HS cohort, patients who underwent TDM exhibited significantly lower mortality rates across all endpoints. Before matching, patients who underwent TDM showed a substantially lower 28‐day mortality rate than those without TDM (24.6% vs. 48.2%, *p* < 0.001) (Table S3). The significant relationship persisted after matching (23.3% vs. 40.8%, *p* = 0.007) (Table 1). Similar reductions were observed in ICU, hospital, 60‐, 90‐, and 365‐day mortality (all *p* < 0.05).

In contrast, among patients with IS, no statistically significant differences in mortality were observed between the TDM and non‐TDM groups after matching (all *p* > 0.05).

### 3.3 Cox Regression

In the HS cohort, TDM was linked to a significantly lower 28‐day mortality risk, and this relationship remained stable both before and after PSM. In the unmatched cohort, TDM was linked to a substantially lower 28‐day mortality risk (HR = 0.50, 95% CI 0.35–0.73, *p* < 0.001), and this beneficial effect remained following PSM (HR = 0.29, 95% CI 0.15–0.56, *p* < 0.001). After controlling for demographic, comorbidity, physiological, and treatment variables, the survival advantage of TDM remained evident, suggesting that the association was independent of confounding influences. Consistent patterns were also observed for secondary outcomes, including ICU, hospital, and long‐term (60‐, 90‐, and 365‐day) mortality (*all p* < 0.05) (Table 2).

**TABLE 2:** Cox regression models (univariate and multivariate) assessing mortality in hemorrhagic stroke patients.

Outcomes	Model 1			Model 2			Model 3		
	HR	95% CI	*p*	HR	95% CI	*p*	HR	95% CI	*p*
*ICU mortality*									
Before PSM	0.33	0.23, 0.48	< 0.001	0.33	0.22, 0.48	< 0.001	0.48	0.29, 0.78	0.003
After PSM	0.48	0.25, 0.94	0.031	0.44	0.23, 0.87	0.018	0.31	0.12, 0.83	0.019
*Hospital mortality*									
Before PSM	0.34	0.23, 0.49	< 0.001	0.35	0.24, 0.51	< 0.001	0.36	0.23, 0.57	< 0.001
After PSM	0.33	0.16, 0.67	0.002	0.32	0.16, 0.65	0.001	0.13	0.05, 0.38	< 0.001
*28 days mortality*									
Before PSM	0.4	0.29, 0.54	< 0.001	0.4	0.29, 0.55	< 0.001	0.5	0.35, 0.73	< 0.001
After PSM	0.51	0.31, 0.85	0.009	0.5	0.30, 0.83	0.008	0.29	0.15, 0.56	< 0.001
*60 days mortality*									
Before PSM	0.42	0.31, 0.56	< 0.001	0.43	0.32, 0.58	< 0.001	0.49	0.34, 0.69	< 0.001
After PSM	0.53	0.33, 0.86	0.01	0.53	0.33, 0.85	0.009	0.36	0.20, 0.64	< 0.001
*90 days mortality*									
Before PSM	0.42	0.32, 0.56	< 0.001	0.43	0.33, 0.58	< 0.001	0.47	0.34, 0.65	< 0.001
After PSM	0.51	0.33, 0.80	0.004	0.51	0.32, 0.80	0.003	0.38	0.22, 0.64	< 0.001
*1 year mortality*									
Before PSM	0.43	0.33, 0.56	< 0.001	0.45	0.35, 0.58	< 0.001	0.5	0.37, 0.68	< 0.001
After PSM	0.57	0.38, 0.84	0.005	0.56	0.38, 0.83	0.004	0.43	0.27, 0.68	< 0.001

*Note:* Model 1: no covariates were adjusted. Model 2: adjusted for age, gender, and race. Model 3: adjusted for age, gender, race, weight, heart rate, respiratory rate, MAP, temperature, SpO_2_, hematocrit, WBC, platelets, Glu, HCO_3_, Ca, potassium, BUN, eGFR, heart failure, coronary artery disease, cardiac arrhythmias, diabetes, hypertension, renal disease, PVD, coagulopathy, SEPSIS, septic shock, vasopressin, mechanical ventilation, RRT, SOFA score, OASIS, CCI, MRSA, accumulated dose, and medication time.

Abbreviations: CI, confidence interval; HR, hazard ratio.

In the IS cohort, vancomycin TDM was not significantly associated with 28‐day mortality in the unmatched analysis (HR = 0.86, 95% CI 0.62–1.10, *p* = 0.361). Consistently, this lack of association persisted after PSM, where TDM remained unrelated to 28‐day survival (HR = 1.06, 95% CI 0.65–1.72, *p* = 0.828). Furthermore, after PSM, no significant associations were observed between TDM and secondary outcomes, including ICU, hospital, and long‐term mortality rates, in the IS population (all *p* > 0.05) (Table 3).

**TABLE 3:** Cox regression models (univariate and multivariate) assessing mortality in ischemic stroke patients.

Outcomes	Model 1			Model 2			Model 3		
	HR	95% CI	*p*	HR	95% CI	*p*	HR	95% CI	*p*
*ICU mortality*									
Before PSM	0.4	0.27, 0.59	< 0.001	0.45	0.30, 0.66	< 0.001	0.47	0.29, 0.76	0.002
After PSM	0.87	0.47, 1.62	0.659	0.86	0.46, 1.61	0.63	0.86	0.38, 1.96	0.721
*Hospital mortality*									
Before PSM	0.53	0.37, 0.76	< 0.001	0.58	0.40, 0.84	0.003	0.5	0.32, 0.76	0.001
After PSM	1.03	0.58, 1.80	0.93	0.99	0.56, 1.74	0.959	0.84	0.42, 1.69	0.619
*28 days mortality*									
Before PSM	0.79	0.60, 1.04	0.098	0.86	0.65, 1.15	0.317	0.86	0.62, 1.19	0.361
After PSM	1.15	0.74, 1.78	0.527	1.13	0.73, 1.75	0.592	1.06	0.65, 1.72	0.828
*60 days mortality*									
Before PSM	0.89	0.69, 1.15	0.382	1.01	0.78, 1.32	0.929	0.95	0.70, 1.29	0.724
After PSM	1.26	0.83, 1.89	0.276	1.23	0.82, 1.86	0.317	1.13	0.71, 1.78	0.606
*90 days mortality*									
Before PSM	0.92	0.72, 1.18	0.521	1.05	0.81, 1.36	0.717	0.94	0.70, 1.26	0.671
After PSM	1.22	0.83, 1.81	0.312	1.2	0.81, 1.78	0.357	1.11	0.72, 1.71	0.631
*1 year mortality*									
Before PSM	0.97	0.77, 1.22	0.785	1.13	0.89, 1.43	0.322	0.96	0.73, 1.26	0.762
After PSM	1.17	0.81, 1.70	0.395	1.16	0.80, 1.67	0.435	1.09	0.73, 1.64	0.678

*Note:* Model 1: no covariates were adjusted. Model 2: adjusted for age, gender, and race. Model 3: adjusted for age, gender, race, weight, heart rate, respiratory rate, MAP, temperature, SpO_2_, hematocrit, WBC, platelets, Glu, HCO_3_, Ca, potassium, BUN, eGFR, heart failure, coronary artery disease, cardiac arrhythmias, diabetes, hypertension, renal disease, PVD, coagulopathy, SEPSIS, septic shock, vasopressin, mechanical ventilation, RRT, SOFA score, OASIS, CCI, MRSA, accumulated dose, and medication time.

Abbreviations: CI, confidence interval; HR, hazard ratio.

### 3.4 Kaplan–Meier Survival

**FIGURE 2:**
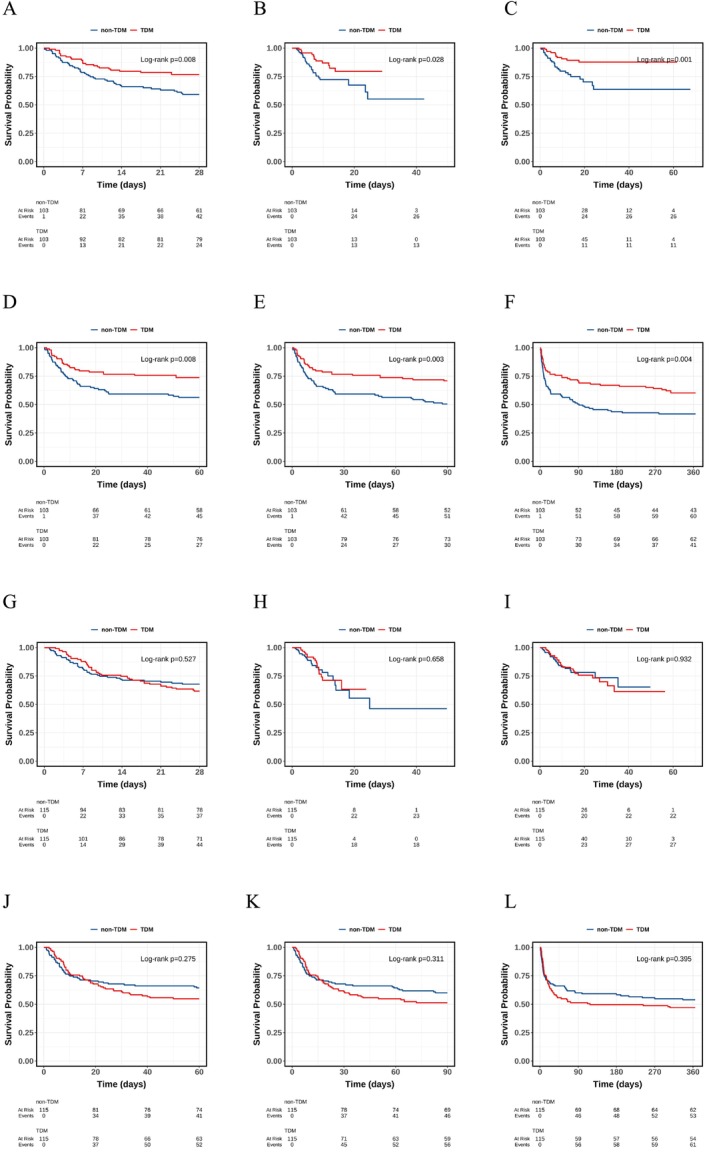
Kaplan–Meier survival curves comparing patients on TDM and non‐TDM after propensity score matching. Survival differences between TDM and non‐TDM patients with hemorrhagic stroke at different time points: (A) 28‐day, (B) ICU, (C) hospital, (D) 60‐day, (E) 90‐day, and (F) 1‐year mortality. Survival differences between TDM and non‐TDM patients with ischemic stroke at different time points: (G) 28‐day, (H) ICU, (I) hospital, (J) 60‐day, (K) 90‐day, and (L) 1‐year mortality.

Survival analyses based on the Kaplan–Meier method were conducted before and after PSM (Figure 2 and Figure S3) revealed results consistent with the regression models. In the subtype analysis, critically ill patients with HS demonstrated a persistent survival benefit associated with TDM in both analyses (all *p* < 0.05). In contrast, among IS patients, unadjusted analyses initially suggested higher ICU and in‐hospital survival rates in the TDM group. However, these apparent benefits were attenuated after PSM, and no significant survival differences remained between the TDM and non‐TDM groups in the matched analysis (all *p* > 0.05).

### 3.5 Subgroup Analyses

**FIGURE 3:**
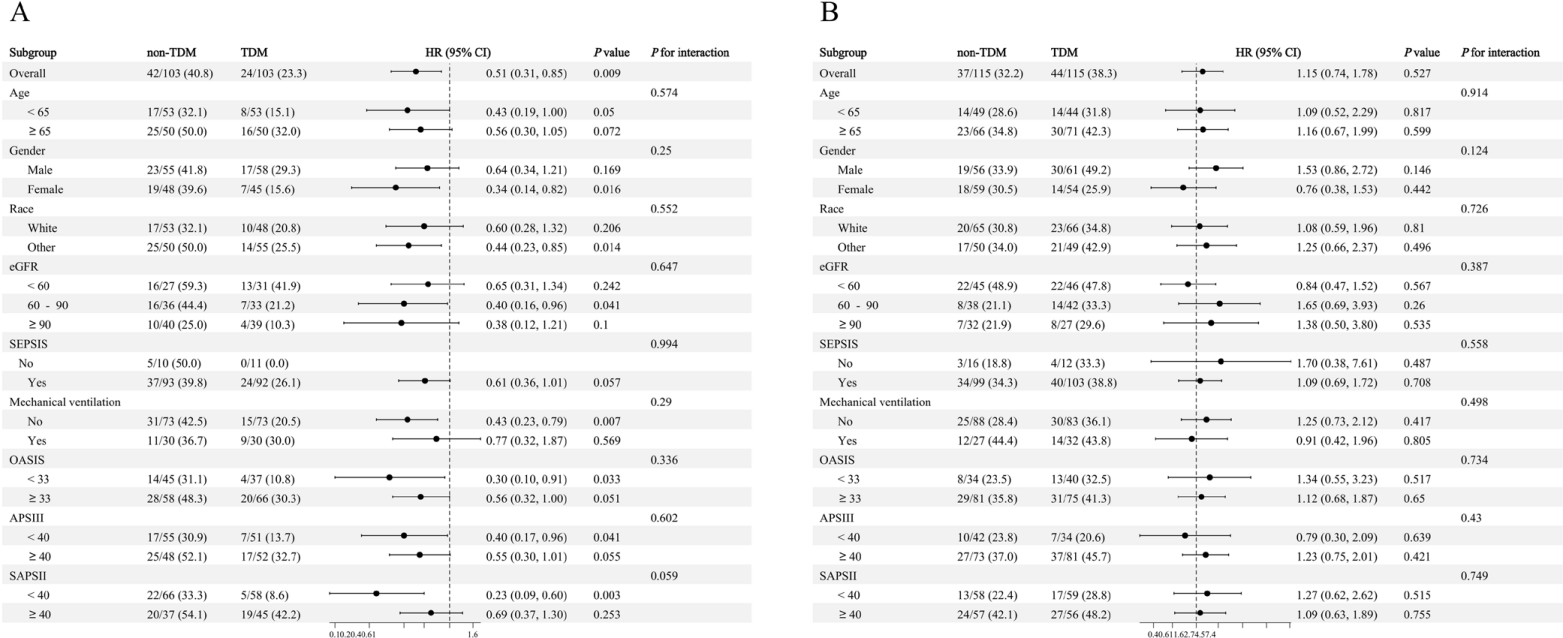
Subgroup analyses of the association between vancomycin therapeutic drug monitoring (TDM) and 28‐day mortality in critically ill stroke patients. (A) Hemorrhagic stroke cohort. (B) Ischemic stroke cohort. Adjusted hazard ratios (HRs) and 95% confidence intervals (CIs) were obtained from Cox proportional hazards models.

Following PSM, subgroup analyses were conducted according to key demographic and clinical variables—such as age, gender, race, eGFR, SEPSIS, mechanical ventilation, and severity indices (OASIS, APS III, SAPS II)—to determine whether TDM was associated with 28‐day mortality. The results were visualized as forest plots (Figure 3).

In the HS cohort, subgroup analyses demonstrated a consistent survival benefit associated with TDM. No significant treatment‐by‐subgroup interactions were detected (all *P* for interaction > 0.05), suggesting that the protective effect of TDM was generally consistent across clinical strata (Figure 3A). Specifically, TDM was significantly associated with reduced 28‐day mortality among female patients (HR = 0.34, 95% CI 0.14–0.82, *p* = 0.016), patients of non‐White race (HR = 0.44, 95% CI 0.23–0.85, *p* = 0.014), and those with mildly impaired to preserved renal function (eGFR 60–90 mL/min/1.73 m^2^; HR = 0.40, 95% CI 0.16–0.96, *p* = 0.041). Furthermore, significant survival improvements were also observed in patients without mechanical ventilation and those with lower disease severity scores (OASIS, APS III, SAPS II) (all *p* < 0.05).

In contrast, for the IS cohort, subgroup analyses revealed no significant associations between TDM and 28‐day mortality across any of the examined categories (Figure 3B). Consistent with the primary analysis, no significant interactions were identified (all *p* for interaction > 0.05), confirming that the lack of survival benefit associated with TDM was uniform across different patient profiles.

### 3.6 Sensitivity Analysis

To rigorously evaluate the robustness of our findings against potential biases—particularly immortal time bias and surveillance bias—five pre‐specified sensitivity analyses were performed on the initial cohort of 1266 patients (543 with HS and 723 with IS). Model 1 excluded patients with a survival time of less than 48 h (retaining 525 HS and 717 IS patients). In the PSM‐adjusted HS cohort, vancomycin TDM remained independently associated with a significant reduction in 28‐day mortality (adjusted HR = 0.50, 95% CI 0.27–0.93, *p* = 0.028). Similarly, Model 2 excluded patients with an intravenous vancomycin treatment duration of less than 48 h (retaining 420 HS and 534 IS patients). Under this stringent condition, the survival benefit of TDM in the matched HS cohort was even more pronounced (adjusted HR = 0.07, 95% CI 0.01–0.36, *p* = 0.001).

When MRSA‐positive cases were excluded (Model 3: retaining 511 HS and 678 IS patients), TDM remained associated with reduced 28‐day mortality in the matched HS cohort (HR = 0.576, 95% CI 0.332–0.998, *p* = 0.049). Likewise, after further adjustment for the overall intensity of care (Model 4: including both total laboratory test count and blood culture frequency), the protective association persisted (HR = 0.58, 95% CI 0.34–0.98, *p* = 0.043). Finally, utilizing a stricter definition of TDM requiring at least two serum concentration measurements (Model 5), the analysis confirmed a significant independent reduction in 28‐day mortality among matched HS patients (adjusted HR = 0.35, 95% CI 0.20–0.62, *p* < 0.001).

In summary, vancomycin TDM consistently demonstrated a significant survival benefit in the HS cohort across all five sensitivity scenarios (Table S4). In contrast, no significant association between TDM and 28‐day mortality was observed in the propensity‐matched IS cohort across any of the sensitivity analyses (all *p* > 0.05). (Table S5).

## 4 Discussion

Using data from the large‐scale MIMIC‐IV critical care database, this study found that vancomycin TDM correlated with reduced short‐ and long‐term mortality in hemorrhagic stroke patients, while no comparable association was identified in those with ischemic stroke. After PSM, baseline characteristics were well balanced, and the association remained robust, suggesting that the survival benefit of TDM in HS patients was independent of potential confounding factors. Subgroup analyses within the HS cohort revealed no significant interaction effects, indicating a consistent protective association across clinical subgroups. Additional sensitivity analyses yielded consistent outcomes, reinforcing the reliability and robustness of the observed associations. Collectively, this study provides the first comprehensive evidence that vancomycin TDM confers significant and sustained survival benefits in critically ill patients with HS. These results highlight the value of incorporating TDM into clinical protocols to enhance personalized antimicrobial management in neurocritical care settings.

Our results align with prior studies in critically ill ICU populations, which have reported that vancomycin TDM is linked to lower mortality rates [27, 28]. However, prior research has mainly focused on general ICU cohorts, and evidence regarding this approach in the neurologically critical subgroup of stroke patients remains limited [27, 28, 36, 37]. To date, no study has systematically evaluated the survival benefits or clinical value of TDM in critically ill patients with stroke. This study provides the first evidence that TDM may be associated with better survival among patients within this distinct clinical population.

Stroke patients exhibit distinct pathophysiological alterations, including stroke‐induced immunosuppression (SIDS) and hemodynamic instability [10, 12, 13]. A heightened systemic inflammatory reaction is frequently observed in patients suffering from HS [29, 38, 39, 40]. TDM plays a crucial role in maintaining an optimal AUC/MIC ratio (400–600 mg·h/L), thereby ensuring bactericidal efficacy while minimizing nephrotoxicity [24].

Type‐specific analyses revealed substantial heterogeneity between stroke subtypes: the survival benefit of TDM was pronounced and consistent among patients with HS but not significant in those with IS. This discrepancy may stem from fundamental differences between the two subtypes in systemic inflammation, immune dysregulation, and renal hemodynamics. A heightened systemic inflammatory reaction is frequently observed in HS [41, 42] and a higher likelihood of augmented renal clearance [43] leading to greater interindividual variability in vancomycin pharmacokinetics, which makes TDM‐guided dose optimization more critical. In contrast, IS patients may exhibit augmented renal clearance (ARC) less consistently [44] and less extreme inflammatory derangement, thus reducing the observable survival benefit of TDM in that subgroup. By dynamically reflecting drug exposure and guiding individualized dose adjustments, TDM helps achieve effective antimicrobial concentrations at the site of infection, improve infection control, promote neurological recovery, and enhance overall survival—particularly in HS patients with enhanced (ERC) or ARC, where its clinical value is especially evident [45, 46, 47]. In addition, clinical practice patterns may further contribute to this divergence. HS patients more frequently undergo neurosurgical procedures such as hematoma evacuation or decompressive craniectomy, which substantially increase the risk of postoperative central nervous system (CNS) infections [48, 49]. Because many of these infections are caused by Gram‐positive organisms, achieving optimal vancomycin exposure becomes particularly important for effective infection control [50, 51, 52, 53]. TDM may therefore play a more prominent role in HS patients by ensuring adequate drug concentrations in the setting of elevated infection risk and altered physiological states.

This study possesses several notable strengths. First, it leveraged the extensive, high‐quality MIMIC‐IV critical care database, which includes a diverse ICU population and provides strong statistical power, thereby enhancing the generalizability of the findings. Second, and most importantly, we applied a rigorous methodological framework to ensure the reliability of our conclusions. Beyond standard multivariable Cox regression and PSM, we implemented a comprehensive series of five pre‐specified sensitivity analyses specifically designed to address immortal time bias and surveillance bias—common pitfalls in observational studies of therapeutic monitoring.

A critical methodological challenge in such studies is the risk that the observed survival benefit might simply reflect the fact that patients must survive long enough to receive TDM (immortal time bias). To strictly rule this out, we conducted sensitivity analyses excluding patients with early mortality (< 48 h) and those with brief vancomycin treatment durations (< 48 h). The persistence of the survival benefit in the HS cohort across these stringent models—particularly in Model 2, where the protective effect was most pronounced—strongly suggests that the primary findings were not driven by early survival bias. Furthermore, the results remained robust after adjusting for the intensity of care (Model 4) and applying a stricter definition of TDM (Model 5), confirming that the association was not confounded by more aggressive medical attention or misclassification. Collectively, this rigorous statistical approach supports the internal validity of our findings and suggests that TDM confers a genuine clinical advantage in hemorrhagic stroke patients.

This investigation is, to our knowledge, the first to comprehensively analyze the clinical relevance of vancomycin TDM in critically ill patients with stroke, including both ischemic and hemorrhagic forms, thereby contributing new perspectives for individualized antibiotic optimization in neurocritical care practice.

Despite its strengths, this study has several limitations that merit consideration. First, owing to its retrospective observational design, residual confounding cannot be fully excluded. Although missingness in vital signs and hematological parameters was low (all < 2%), multiple imputation may introduce minor uncertainty. Given the limited extent of missingness, the impact of imputation on effect estimates is likely minimal.

Second, infection‐related and microbiological data were incompletely captured. Many patients lacked culture‐positive results, microbiological timestamps could not be reliably aligned with vancomycin initiation, and ICD‐based diagnoses lacked sufficient granularity for systematic stratification. Consequently, pathogen‐ or resistance‐specific analyses and verification of vancomycin indications at the individual level were not feasible. Moreover, most patients received concomitant empirical broad‐spectrum antibiotics, but heterogeneity and overlapping timing of regimens precluded consistent statistical adjustment. These factors may introduce unmeasured confounding related to infection severity and treatment heterogeneity.

Third, renal function was estimated using the CKD‐EPI 2021 equation because continuous eGFR records were unavailable in MIMIC‐IV. Although comparison analyses suggested minimal deviation from derived values, reliance on calculated estimates may still introduce slight imprecision.

Fourth, although CNS infections may contribute to the differential survival benefit of vancomycin TDM in hemorrhagic stroke, the limited number of clearly documented cases precluded meaningful subgroup or adjusted analyses.

Fifth, despite leveraging a large, high‐quality critical care cohort, this study relied exclusively on the single‐center MIMIC‐IV database. The absence of external validation limits assessment of generalizability across institutions, patient populations, and stewardship environments.

Finally, vancomycin exposure was assessed using cumulative dose and treatment duration, while other key pharmacokinetic and safety parameters—including initiation timing, individual dosing, peak concentrations, and nephrotoxicity—could not be systematically evaluated. As a result, dose–response variability and safety profiles may be underestimated.

Considering the retrospective and single‐center design of the present study, future investigations are warranted to address these limitations and enhance the translational relevance of the findings. First, prospective, multicenter studies with external validation are warranted to more rigorously examine the association between vancomycin TDM and stroke outcomes. Given the observational nature of the present study, future investigations using standardized TDM protocols and predefined analytic frameworks will be essential to better clarify the robustness and generalizability of these findings. Second, CNS infections may play an important role in modulating the effectiveness of vancomycin TDM in hemorrhagic stroke patients. Future prospective studies incorporating comprehensive neurosurgical infection data and standardized diagnostic criteria are required to clarify the clinical value of TDM in hemorrhagic stroke patients with CNS infections. Third, the development of real‐time TDM systems integrating electronic health records, Bayesian pharmacokinetic modeling, and machine learning may enable more granular characterization of vancomycin exposure [54]. In addition, more research is needed to clarify how TDM interacts with systemic inflammation, immune responses, and stroke subtype–related pathophysiological processes, thereby shedding light on the pharmacokinetic mechanisms that influence neurological outcomes. Finally, applying the concept of TDM to additional key antimicrobials in neurocritical care may help develop a broader and more precision‐oriented framework for infection control and antibiotic stewardship.

## 5 Conclusion

Analysis of the large MIMIC‐IV critical care database indicated that vancomycin TDM was independently linked to lower short‐ and long‐term mortality in critically ill patients with HS. This relationship persisted after controlling for multiple covariates, PSM, and comprehensive sensitivity analyses, suggesting that the observed survival advantage of TDM was not confounded by disease severity or other clinical factors. In contrast, no significant survival advantage was observed among patients with IS. Subgroup analyses within the HS cohort did not identify any significant interaction effects, supporting the robustness and consistency of the association between vancomycin TDM and reduced mortality across the examined clinical subgroups. In summary, the present analysis highlights TDM as a promising component of precision antimicrobial stewardship that may improve survival among critically ill patients with HS. However, additional research is needed to determine whether similar benefits extend to those with IS.

## Author Contributions

H.S.: Conceived the study design and finalized the manuscript. J.F.: performed the data extraction and initial analysis. H.Z.: Designed the figures and tables. M.Z. and J.Y.: assisted in the data cleaning, data proofreading, and statistical analysis. J.G., L.L., and Y.H.: prepared the initial manuscript draft. C.L., H.C., and J.J.: contributed to figure plotting. S.L.: Directed the study. X.W.: Directed the study and provided funds. All authors finalized the manuscript, refined the article, and contributed to the final manuscript and have approved it for publication.

## Funding

The present study was funded by The Special Fund Research Projects of The Fourth Affiliated Hospital of Harbin Medical University (No. HYDSYTB202232), The Torch Plan Surface Project of The Fourth Affiliated Hospital of Harbin Medical University (No. HYDSYHJ201902), and The Horizontal Research Project on the Role of LncRNAs and PAK7 in Brain Glioma Cell Proliferation and Temozolomide‐Resistant Glioma (2020–2024).

## Ethics Statement

This study was conducted using the publicly available, de‐identified MIMIC‐IV (v3.1) database under a data use agreement. The use of these data was approved by the Institutional Review Boards of the Massachusetts Institute of Technology and Beth Israel Deaconess Medical Center, with a waiver of informed consent due to the retrospective and fully de‐identified nature of the data. The investigator (Hongbo San; CITI Program Certificate ID: 67554259) completed the required human‐subjects research training and obtained authorized access to the database.

## Consent

The authors have nothing to report.

## Conflicts of Interest

The authors declare no conflicts of interest.

## Supporting information


**Data S1:** cns70799‐sup‐0001‐FigureS1‐S3.pdf.

**Data S2:** cns70799‐sup‐0002‐TableS1‐S5.xlsx.

## Data Availability

Research data are not shared.
