# Supplementary material for: Prognostic Implications of Vancomycin Therapeutic Drug Monitoring for Critically Ill Stroke Patients: Evidence From a Subtype‐Oriented Analysis
Source: CNS Neurosci Ther. 2026 Feb 19;32(2):e70799. doi: 10.1002/cns.70799 (PMC12918411; doi:10.1002/cns.70799)
Supplement: Supplementary file 1 — Data S1: cns70799‐sup‐0001‐FigureS1‐S3.pdf. [file CNS-32-e70799-s001.pdf]

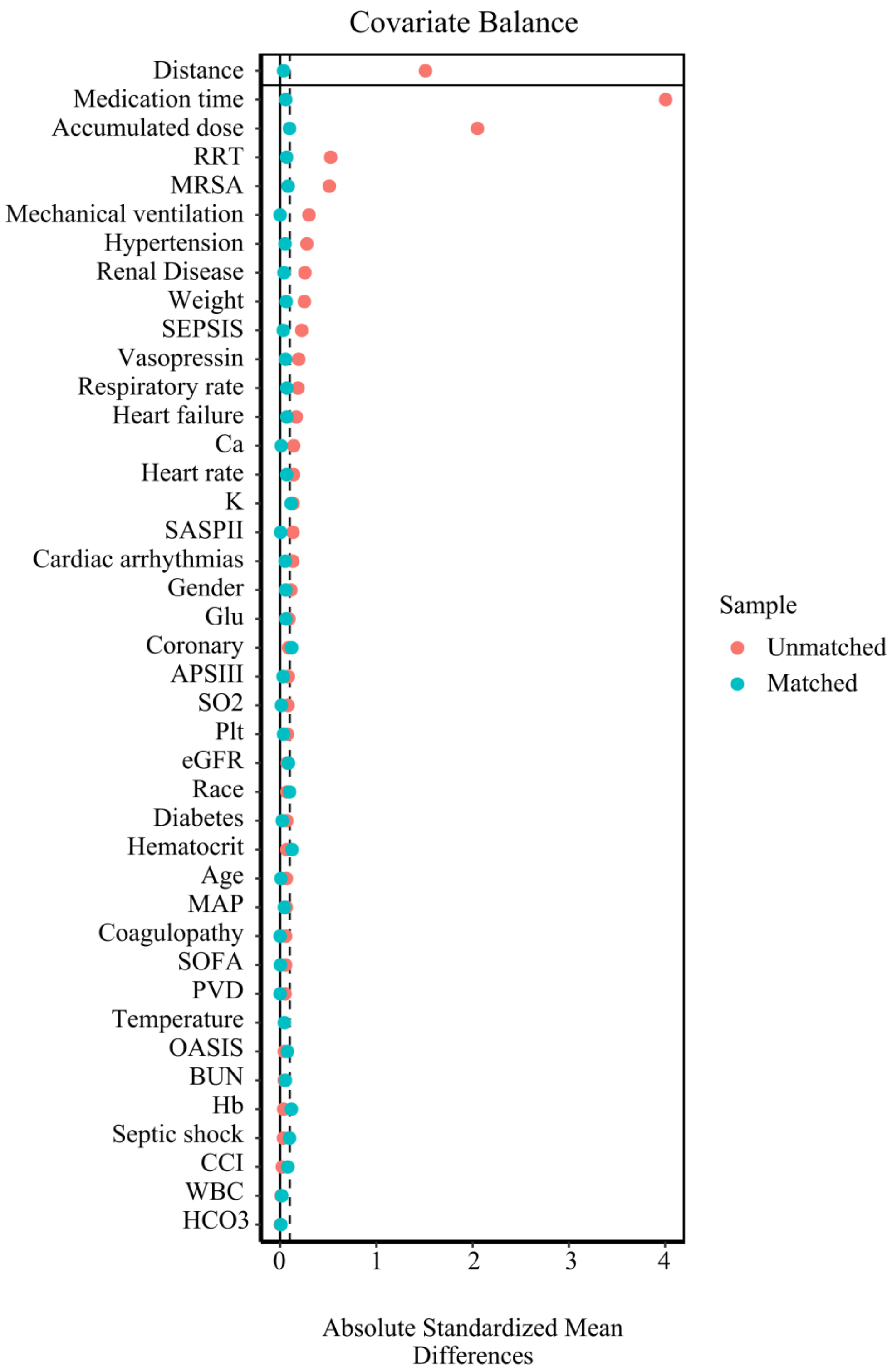

Supplementary Figure S1： Absolute standardized differences of covariates between the TDM and non-TDM groups before and after propensity score matching in patients with hemorrhagic stroke.

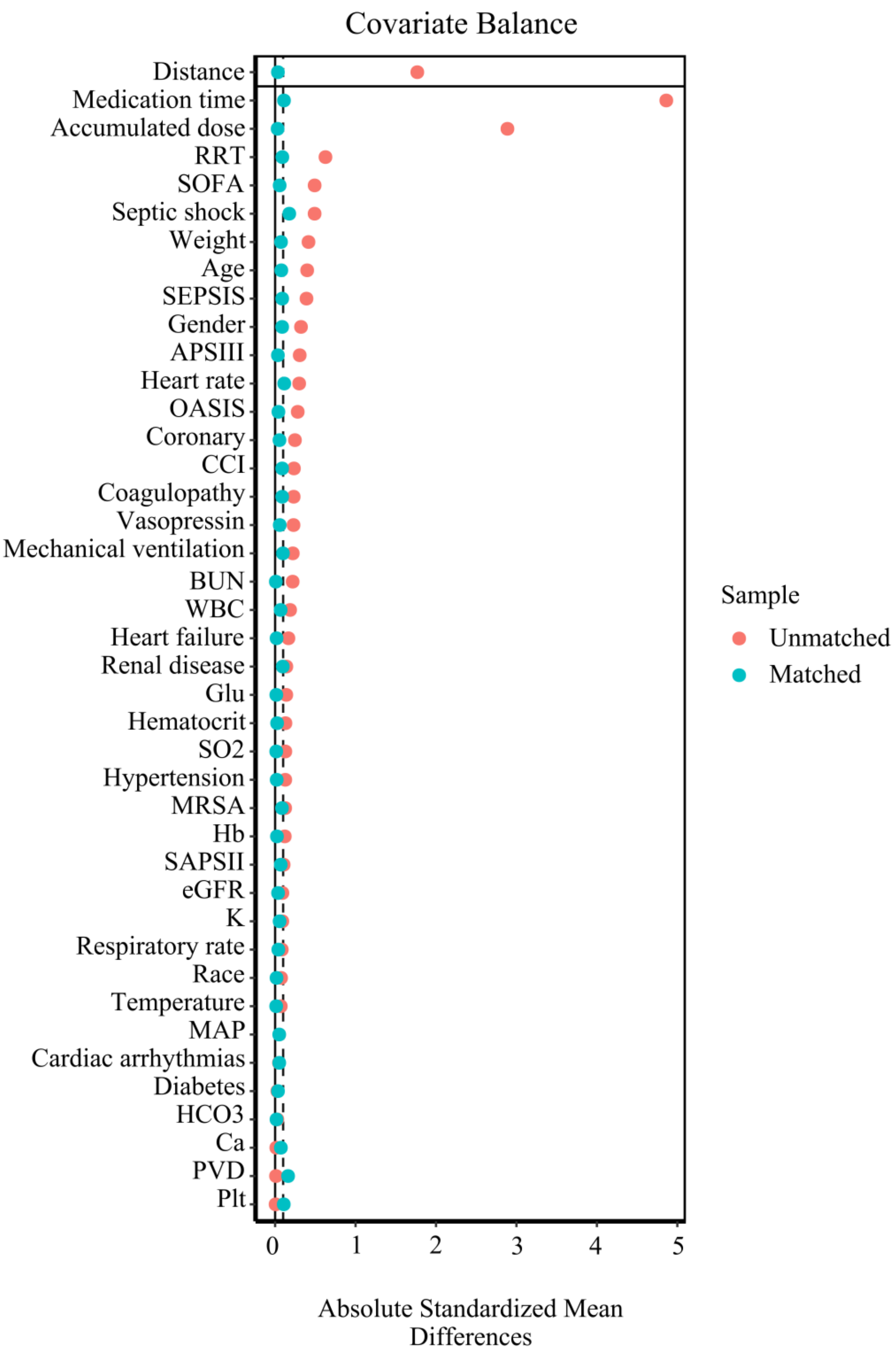

Supplementary Figure S2: Absolute standardized differences of covariates between the TDM and non-TDM groups before and after propensity score matching in patients with ischemic stroke.

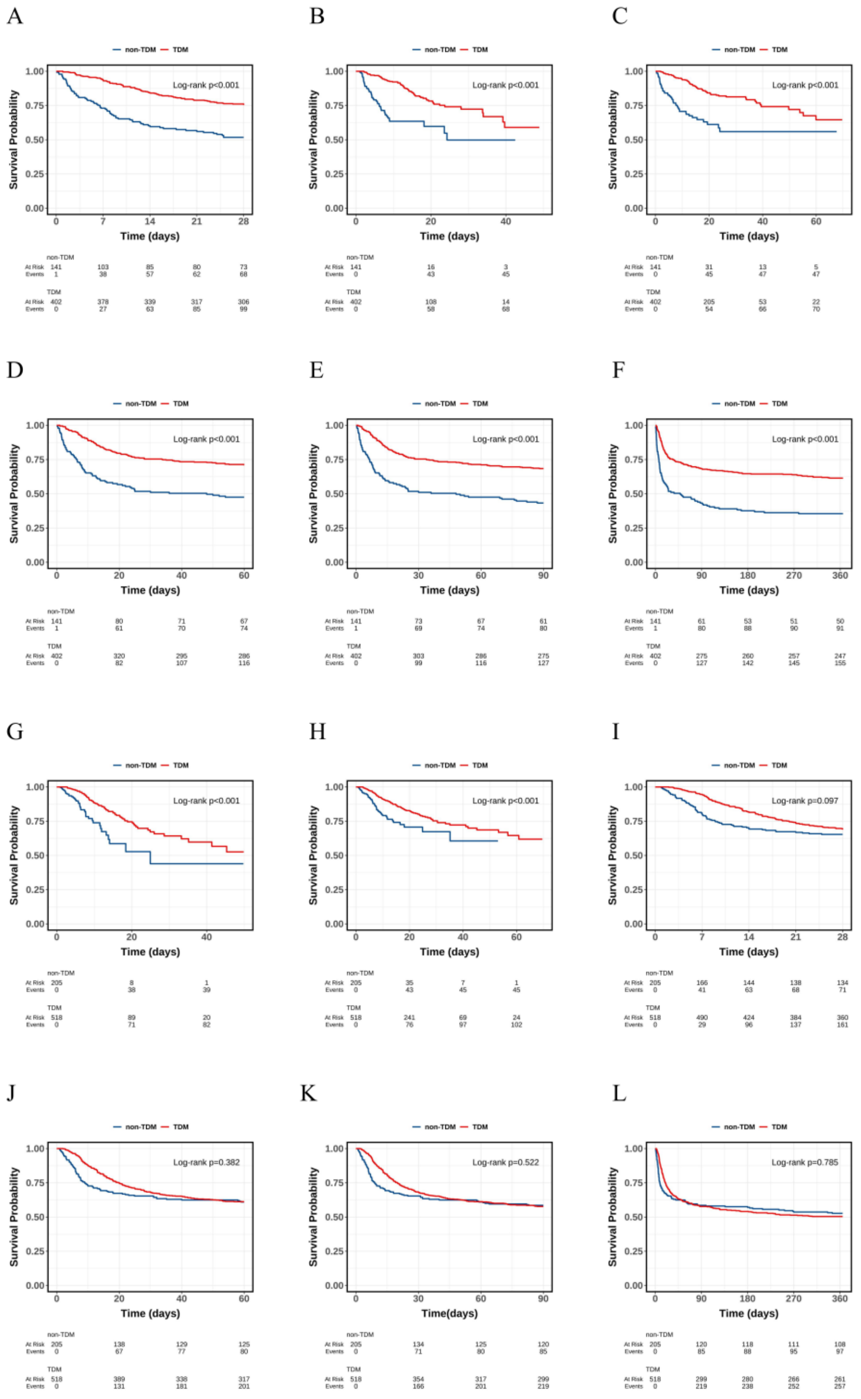

Supplementary Figure S3: Kaplan–Meier survival curves comparing patients TDM and non-TDM before propensity score matching. (A–F) Survival differences between TDM and non-TDM patients with hemorrhagic stroke at different time points: (A) 28-day, (B) ICU, (C) hospital, (D) 60-day, (E) 90-day, and (F) 1-year mortality. (G–L) Survival differences between TDM and non-TDM patients with ischemic stroke at different time points: (G) 28-day, (H) ICU, (I) hospital, (J) 60-day, (K) 90-day, and (L) 1-year mortality.
